# An eukaryotic elongation factor 2 from *Medicago falcata* (*MfEF2*) confers cold tolerance

**DOI:** 10.1186/s12870-019-1826-7

**Published:** 2019-05-27

**Authors:** Haifan Shi, Sijian He, Xueying He, Shaoyun Lu, Zhenfei Guo

**Affiliations:** 1 College of Grassland Science, Nanjing Agricultural University, Nanjing, 210095 China; 2 State Key Laboratory for Conservation and Utilization of Subtropical Agro-bioresources, College of Life Sciences, Guangdong Engineering Research Center for Grassland Science, South China Agricultural University, Guangzhou, 510642 China

**Keywords:** Cold stress, iTRAQ, *Medicago falcata*, MfEF2, Photosynthesis, Splicesome

## Abstract

**Background:**

An eukaryotic translation elongation factor-2 (eEF-2) plays an important role in protein synthesis, however, investigation on its role in abiotic stress responses is limited. A cold responsive *eEF2* named as *MfEF2* was isolated from yellow-flowered alfalfa [*Medicago sativa subsp. falcata* (L.) Arcang, thereafter *M. falcata*], a forage legume with great cold tolerance, and transgenic tobacco (*Nicotiana tabacum* L.) plants overexpressing *MfEF2* were analyzed in cold tolerance and proteomic profiling was conducted under low temperature in this study.

**Results:**

*MfEF2* transcript was induced and peaked at 24 h and remained at the high level during cold treatment up to 96 h. Overexpression of *MfEF2* in trasngenic tobacco plants resulted in enhanced cold tolerance. Compared to the wild type, transgenic plants showed higher survival rate after freezing treatment, higher levels of net photosynthetic rate (*A*), maximum photochemical efciency of photosystem (PS) II (*F*_v_/*F*_m_) and nonphotochemical quenching (*NPQ*) and lower levels of ion leakage and reactive oxygen species (ROS) production after chilling treatment. iTRAQ-based quantitative proteomic analysis identified 336 differentially expressed proteins (DEPs) from leaves of one transgenic line versus the wild type after chilling treatment for 48 h. GO and KEGG enrichment were conducted for analysis of the major biological process, cellular component, molecular function, and pathways of the DEPs involving in. It is interesting that many down-regulated DEPs were grouped into “photosynthesis” and “photosynthesis-antenna”, such as subunits of PSI and PSII as well as light harvesting chlorophyll protein complex (LHC), while many up-regulated DEPs were grouped into “spliceosome”.

**Conclusions:**

The results suggest that MfEF2 confers cold tolerance through regulating hundreds of proteins synthesis under low temperature conditions. The elevated cold tolerance in *MfEF2* transgenic plants was associated with downregulation of the subunits of PSI and PSII as well as LHC, which leads to reduced capacity for capturing sunlight and ROS production for protection of plants, and upregulation of proteins involving in splicesome, which promotes alternative splicing of pre-mRNA under low temperature.

**Electronic supplementary material:**

The online version of this article (10.1186/s12870-019-1826-7) contains supplementary material, which is available to authorized users.

## Background

Low temperature is one of the major abiotic stresses that limits the distribution and productivity of crops worldwide. Many plants from temperate regions can enhance their freezing tolerance after exposure to low, non-freezing temperatures, the process was known as cold acclimation [1]. Extensive changes occur ranging from gene expression to biochemical, physiological and metabolic processes during cold acclimation [2–4]. Numerous cold-related genes such as CRT binding factor (CBF) and cold regulated (COR) genes have been identified [5–7]. Osmolytes and cryoprotectants, such as soluble sugars (saccharose, raffinose, trehalose), sugar alcohols (sorbitol, ribitol, inositol) and nitrogenous compounds (proline), are accumulated, and scavenging of reactive oxygen species (ROS) is activated during cold acclimation [8, 9].

*Medicago falcata* is widely distributed in the cold areas of Russia, Mongolia, Scandinavia and northern China, with great cold and drought tolerance and similar genetic background to alfalfa [10, 11]. It is an important gene pool for alfalfa breeding and resulted in significant heterosis for biomass yield [12, 13]. Thus it is important to understand its mechanisms in cold tolerance and to discover new genes using for improvement of cold tolerance in crops. A serous of cold responsive genes in *M. falcata*, such as *MfMIPS1* [11], *MfGolS1* [14], *MfINT-like* [15], *MfSAMS1* [16], *MfTIL1* [17], and *MfPIP2–7* [18], and *MfERF* [19], have been documented to be associated with cold tolerance. An eukaryotic elongation factor 2 encoding gene (*eEF2*) was found as cold responsive genes in the cDNA library of *M. falcata* using suppression subtractive hybridization (SSH) [20], implying that *eEF2* might be associated with cold tolerance in *M. falcata*.

Elongation factors play an important role in translation. Translation is a three-stage process comprising initiation, elongation and termination. In the peptide elongation phase of protein synthesis, elongation factor 1A transports amino acylated tRNA to the ribosome acceptor A-site, where peptidyl-tRNA is formatted, catalyzed by peptidyl transferase [21]. The pre-translocational state of the ribosome is the substrate of the GTPase EF2, which leaves a vacant A-site to allow the next aminoacyl-tRNA to enter for starting a new cycle of peptide formation [21]. Eukaryotic translation elongation factor-2 was found to regulate rhythmic protein accumulation in *Neurospora crassa* [22]. Suppression of elongation is responsible for the significant reduction in global protein synthesis in mammalian cells [23]. Even though eEF2 plays an important role in protein synthesis, investigation on its role in abiotic stress responses is limited. An early study in *Arabidopsis* suggested that eEF2 is associated with plant cold tolerance. One point mutation in the conserved residue Cys495 of EF2 protein in *Arabidopsis* mutant *los1–1* blocks low temperature-induced transcription of cold-responsive genes and reduces the capacity of plants to develop freezing tolerance. Protein synthesis in *los1–1* mutant is impaired at low temperature [24]. However, it is unknown whether cold tolerance is altered in transgenic plants overexpressing *eEF2* gene.

In this study, a coding sequence of *MfEF2* was cloned from *M. falcata*, and transgenic tobacco plants overexpressing *MfEF2* were generated and analyzed. We demonstrated that MfEF2 plays an important role in plant tolerance to cold stress.

## Results

### Cloning and characterization of *MfEF2*

**Fig. 1:**
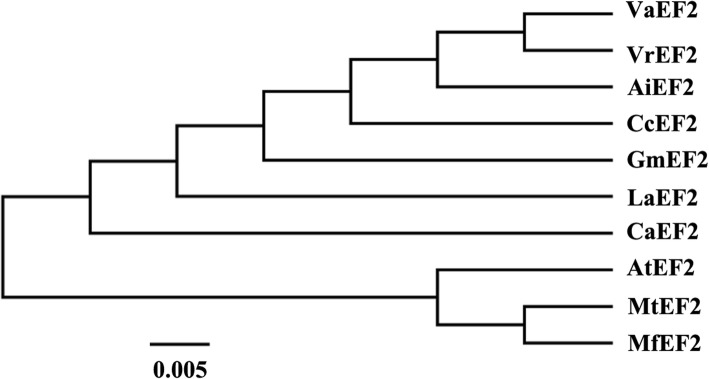
Phylogenetic analysisof MfEF2 with other plant EF2s. The EF2 accession numbers and the specises include are VaEF2 (XP_017424963.1, *Vigna angularis*), VrEF2 (XP_014501194.1, *Vigna radiate*), AiEF2 (XP_016174723.1, *Arachis ipaensis*), CcEF2 (XP_020233398.1, *Cajanus cajan*), GmEF2 (XP_003546795.1, *Glycine max*), LaEF2 (XP_019455730, *Lupinus angustifolius*), CaEF2 (XP_004488812.1, *Cicer arietnum*), AtEF2 (NP_849818.1, *Arabidopsis thaliana*), MtEF2 (XP_013467256.1, *Medicago truncatula*). The bar in represents the branch length equivalent to 0.05 amino acid changes per residue

For analysis of MfEF2 function, a 2532 bp of open reading frame (ORF) of *MfEF2* (accession no. MK125495) was cloned from leaves of cold-treated *M. falcata* plants by RT-PCR. It encodes a peptide of 843 amino acids with an estimated molecular mass of 94.25 kDa and an isoelectric point (pI) of 5.89. Phylogenetic analysis on EF2 from legumes and *Arabidopsis* showed that MfEF2 had high similarity with other plant EF2s (Fig. 1), indicating that EF2s are highly conserved evolutionarily.

### Response of *MfEF2* expression to cold

**Fig. 2:**
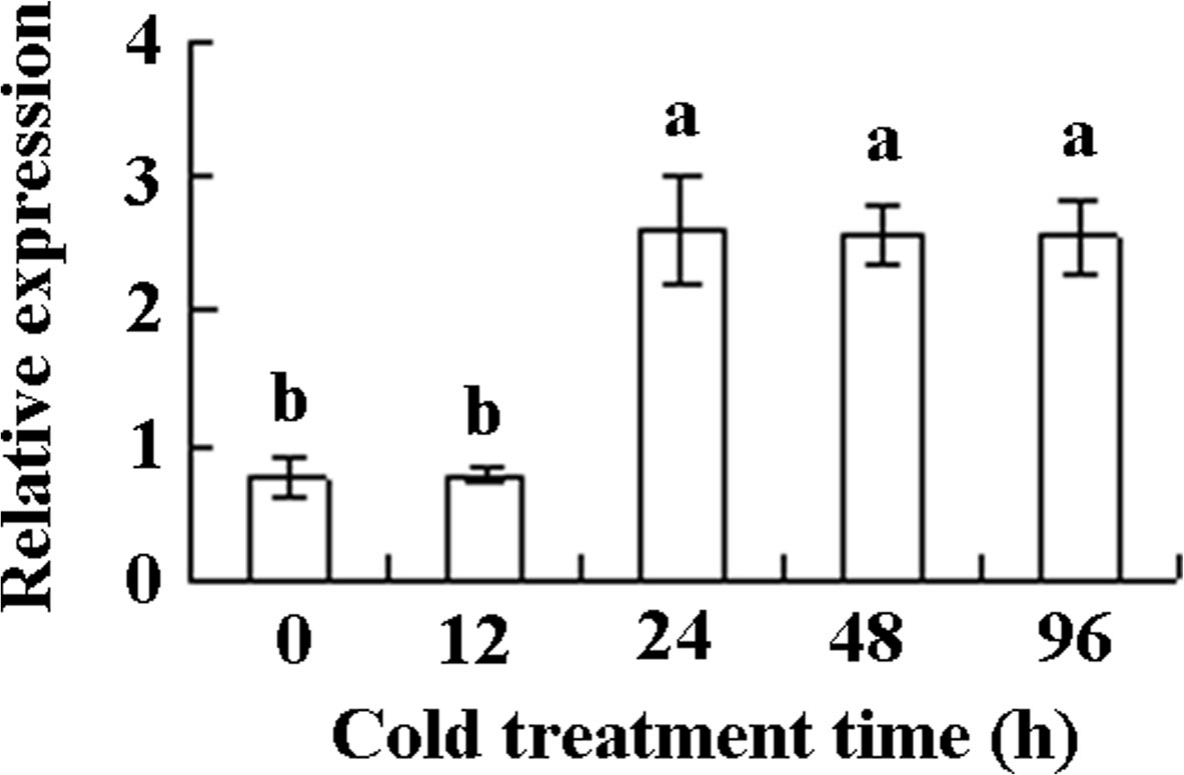
*MfEF2* transcript in response to low temperature. Mature leaves were sampled from pot plants treated in growth chamber at 5 °C. *MfEF2* trasncript was determined using qRT-PCR, and *actin1* was used as reference gene to normalize the amount of template. Means of three independent samples and standard errors are presented; the same letter above the column indicates no significant difference at *P* < 0.05

Transcript levels of *MfEF2* in response to cold was detected using qRT-PCR. The data showed that *MfEF2* transcript was induced by 3.5-fold after 24 to 96 h of cold treatment, while no induction was observed within 12 h of treatment (Fig. 2). The result implied that *MfEF2* expression might be associated with cold tolerance.

### Analysis of transgenic tobacco plants overexpressing *MfEF2*

**Fig. 3:**
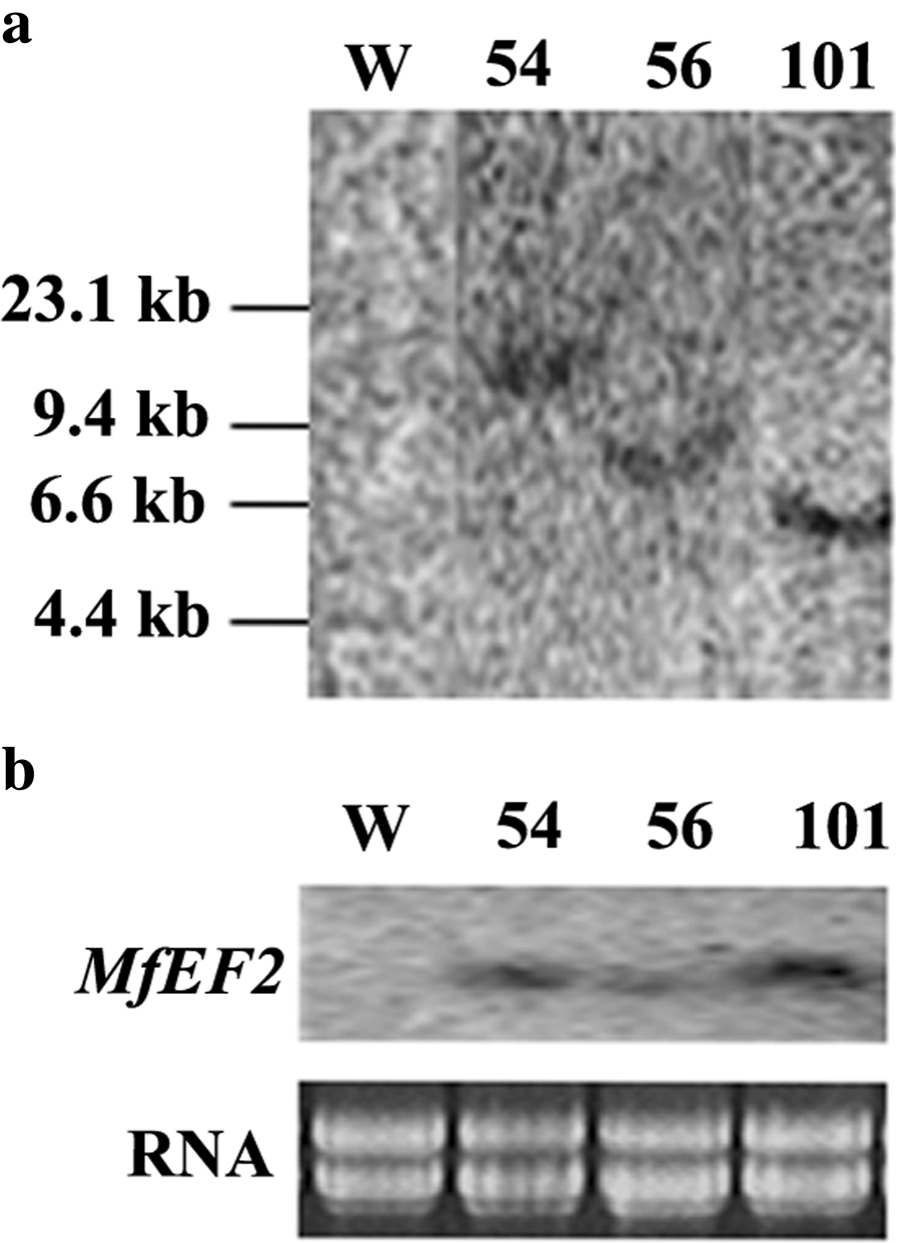
Analysis of the transgenic tobacco plants overexpressing *MfEF2.* Ten micrograms of genomic DNA from each plant line was digested with *Xba* I for DNA blot hybridization (**a**). Twenty micrograms of total RNA was used for RNA blot hybridization (**b**)

To confirm the function of *MfEF2* associated with cold tolerance, transgenic tobacco plants were produced by overexpressing *MfEF2* that was driven by CaMV 35S promoter. Homozygous transgenic plants were harvested by selection with kanamycin resitance from in combination with PCR assay of *MfEF2*. Three homozygous lines (54, 56 and 101) were chosen for analysis of DNA hybridization. Compared to no cross-hybridization signal in the wild type plants, *MfEF2* was one hybridization signal was observed in each transgenic line (Fig. 3a), indicating that *MfEF2* was integrated into the genomes of transgenic tobacco as one transgenic copy. Compared to the wild type, *MfEF2* transcript could be detected in transgenic lines by RNA hybridization (Fig. 3b), indicating that *MfEF2* was expressed in transgenic plants.

### Analysis of freezing and chilling tolerance in transgenic plants

**Fig. 4:**
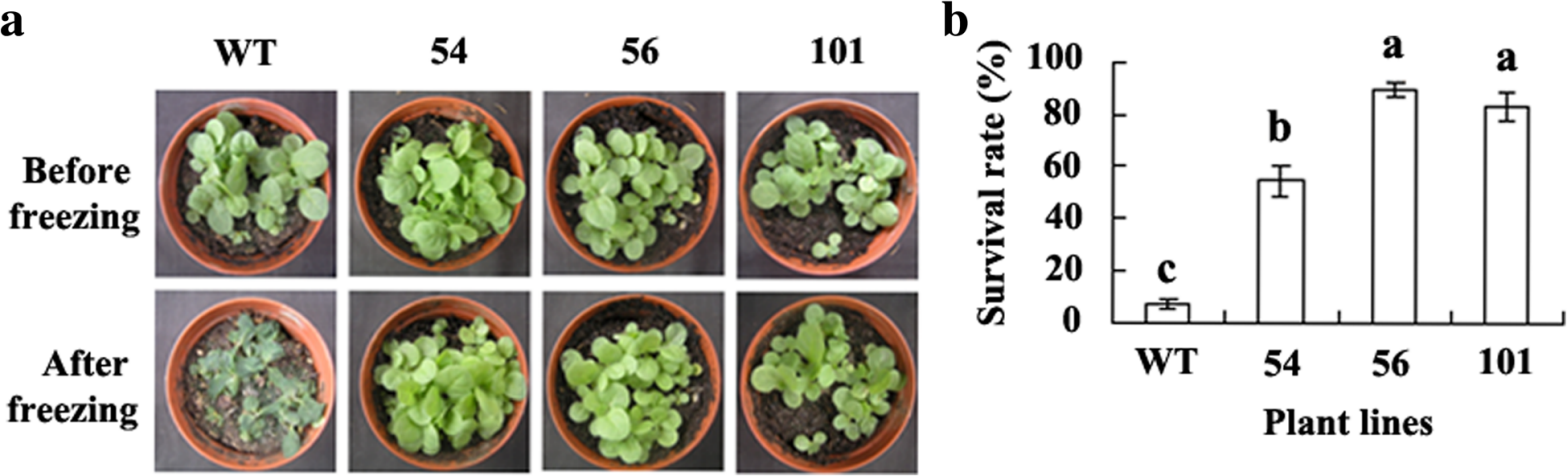
Freezing tolerance of transgenic tobacco plants overexpressing *MfEF2* in comparison with the wild type (WT). Photography was taken before freezing treatment and 7 d of recovery at room temperature after freezing treatment at − 3 °C for 6 h (**a**), followed by calculating survival rate (**b**)

Freezing tolerance was evaluated based on survival rate after exposure to − 3 °C for 10 h. Most of the wild type plants were dead after freezing with a survival rate of 7%, while most of transgenic plants were recovered with survival rate of 54, 90, and 83% in lines 54, 56, and 101, respectively (Fig. 4a, b).

**Fig. 5:**
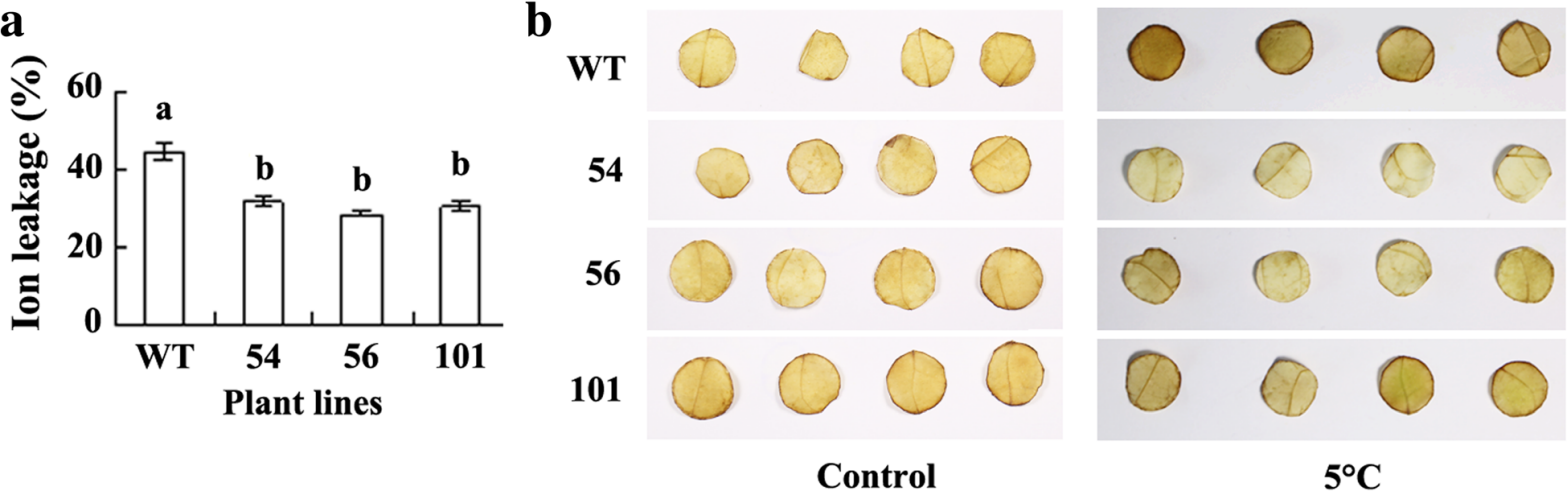
Ion leakage and H_2_O_2_ accumulation in transgenic tobacco plants overexpressing *MfEF2* in comparison with the wild type (WT) after chilling treatment*.* Ion leakage (**a**) and H_2_O_2_ (**b**) were determined 3 d after plants were treated at 2 °C. Means of three independent samples and standard errors are presented; the same letter above the column indicates no significant difference at *P* < 0.05

**Fig. 6:**
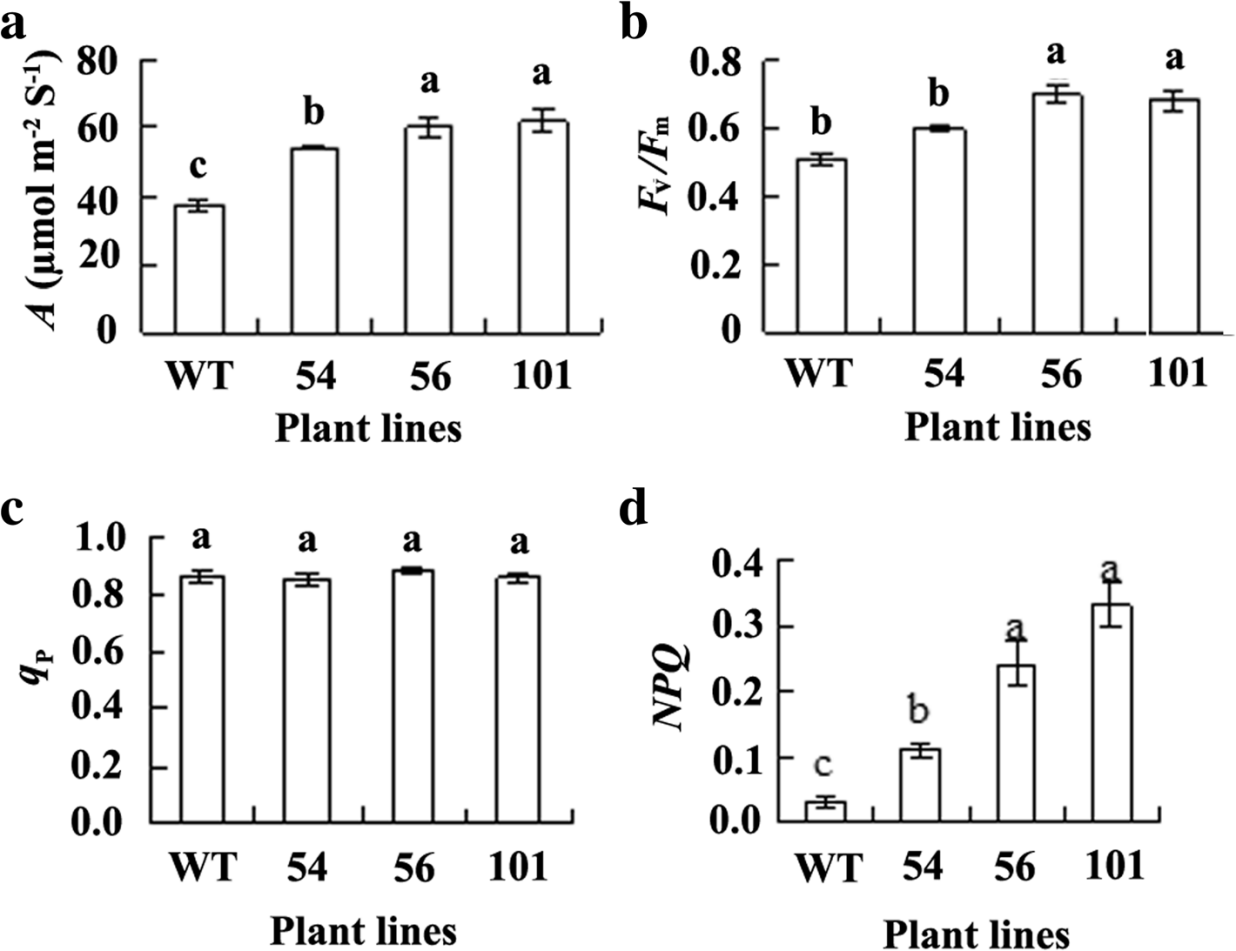
Net photosynthetic rate (*A*) and chlorophyll a fluorescence index in transgenic tobacco plants overexpressing *MfEF2* in comparison with the wild type (WT) after chilling treatment*. A* (**a**), *F*_v_/*F*_m_ (**b**), *q*_p_ (**c**) and *NPQ* (**d**) were determined 3 d after plants were treated at 2 °C. Means of three independent samples and standard errors are presented; the same letter above the column indicates no significant difference at *P* < 0.05

Chilling tolerance was evaluated based on ion leakage, ROS accumulation, and photosynthesis in response to low temperature. The data showed that lower levels of ion leakage and H_2_O_2_ accumulation were observed in transgenic plants than in the wild type after 3 d of chilling treatment at 2 °C, while low level of H_2_O_2_ was detected under control condition in all plant lines (Fig. 5a, b). Higher levels of *A* and *F*_v_*/F*_m_ were observed in transgenic plants than in the wild type after 3 d of chilling treatment at 2 °C (Fig. 6a, b). The results indicated that transgenic plants had increased freezing and chilling tolerance. Compared to no difference in *q*_p_ between transgenic plants and the wild type (Fig. 6c), higher level of *NPQ* was observed in transgenic lines after chilling treatment (Fig. 6d).

### Primary proteomic data analysis and protein identification

Given that EF2 functions in the peptide elongation phase of protein synthesis, an iTRAQ analysis was conducted to observe the proteomic difference in cold-treated leaves between a transgenic tobacco line (54) and the wild type. iTRAQ analysis of tobacco leaves generated 359,430 MS/MS spectra. 23,855 spectra could be matched to unique peptide and 16,261 were considered as unique spectra. A total of 13,128 peptides and 2939 proteins were identified (Additional file 1: Figure S1; Additional file 2: Table S1). Most of the proteins (66.2%) were distributed among 20 to 80 kDa, and small proteins with less than 10 kDa were also detected (Additional file 3: Figure S2). The mass spectrometry proteomics data have been deposited to the ProteomeXchange Consortium (http://proteomecentral.proteomexchange.org) via the iProX partner repository [25] with the dataset identifier PXD011822.

### Functional classification of the differentially expressed proteins

**Fig. 7:**
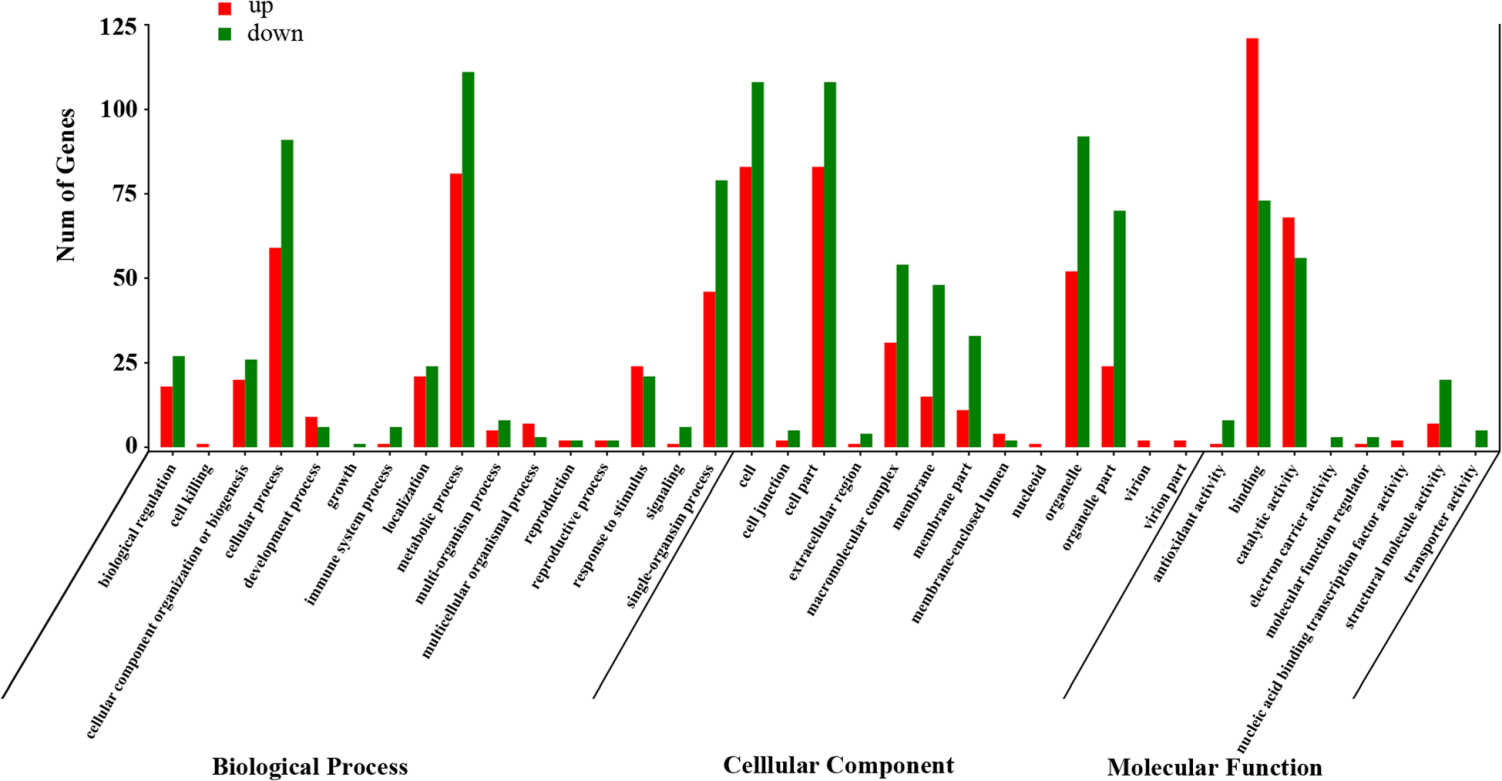
Gene Ontology (GO) annotation terms of differentially expressed proteins (DEPs) at GO level 2 after chilling treatment

The differentially expressed proteins (DEPs) between transgenic plant and the wild type were screened based on the standard criteria: fold-change > 1.2 and *P*-value < 0.05. The data showed that 336 proteins including 181 up-regulated proteins and 155 down-regulated proteins were differentially expressed in transgenic tobacco as compared with the wild type after chilling treatment (Additional file 4: Table S2). Two hundred and two DEPs were classified into biological process, 269 were classified into molecular function, and 240 were classified into cellular component at GO level 2. Compared to other groups, most of the DEPs were grouped to cell process, metabolic process, single-organism process among biological process proteins; most of the DEPs were grouped to cell, cell-part, organelle, and organelle-part among cellular component proteins, and most of the DEPs were grouped to binding and catalytic activity among molecular function proteins (Fig. 7).

**Fig. 8:**
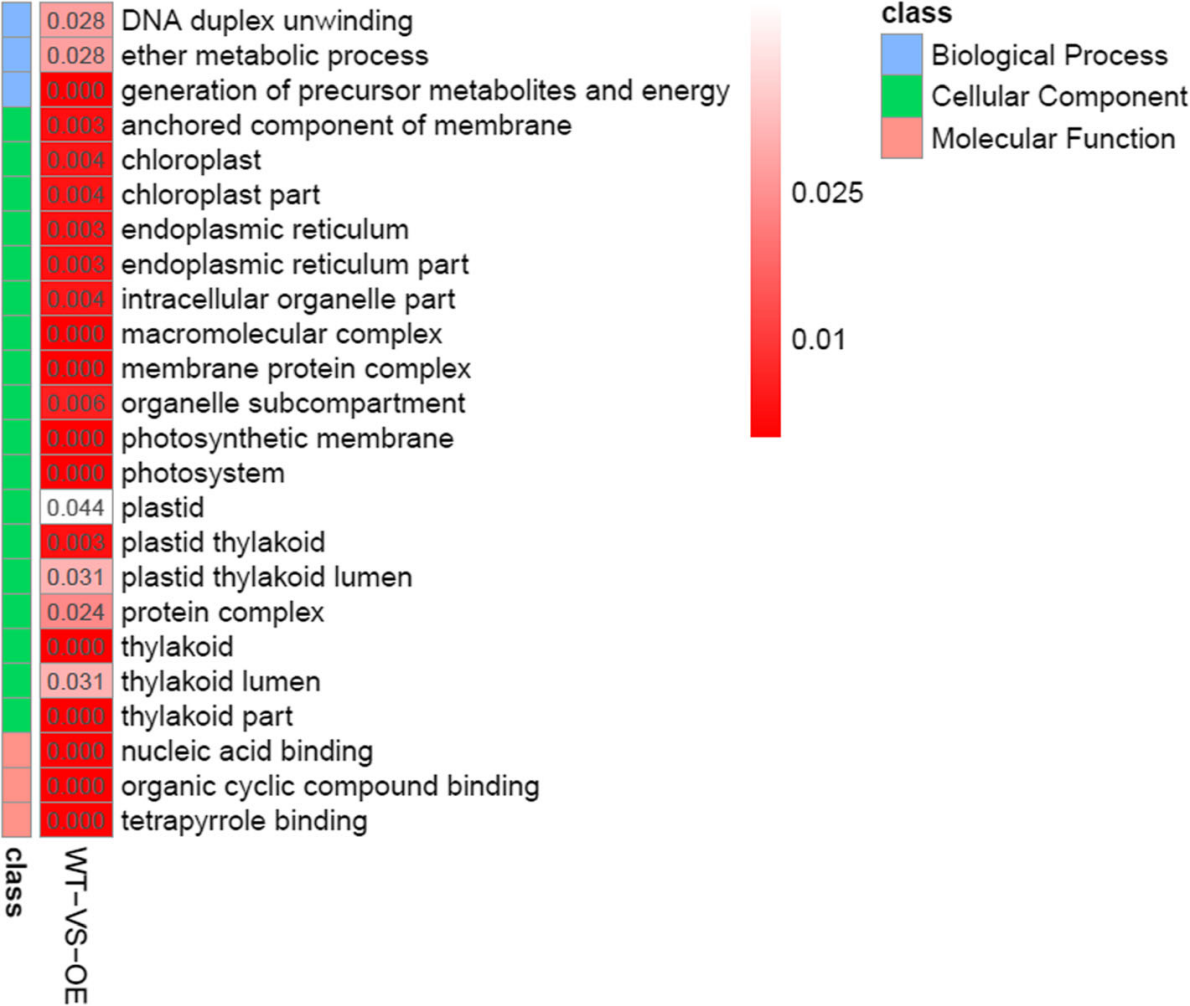
GO enrichment analysis of DEPs after chilling treatment

GO enrichment analysis of DEPs at high level were conducted to obtain more detail functional categorization. The data showed that the DEPs in biological process were mainly subgrouped to “generation of precursor metabolites and energy”, “DNA duplex unwinding” and “ether metabolic process”. The DEPs in molecular function were mainly subgrouped to “nucleic acid binding”, “organic cyclic compound binding” and “tetrapyrrole binding”. The DEPs in cellular component were subgrouped to “anchored component of membrane”, “chloroplast”, “chloroplast part”, “endoplasmic reticulum”, “endoplasmic reticulum part”, “intracellular organelle part”, “macromolecular complex”, “membrane protein complex”, “organelle subcompartment”, “photosynthetic membrane”, “photosystem”, “plastid”, “plastid thylakoid”, “plastid thylakoid lumen”, “protein complex”, “thylakoid”, “thylakoid lumen” and “thylakoid part” (Fig. 8). It is interesting that many DEPs were focused on subcellular localization associated with photosynthesis, indicating expression of MfEF2 was involved in regulation on photosynthesis.

**Fig. 9:**
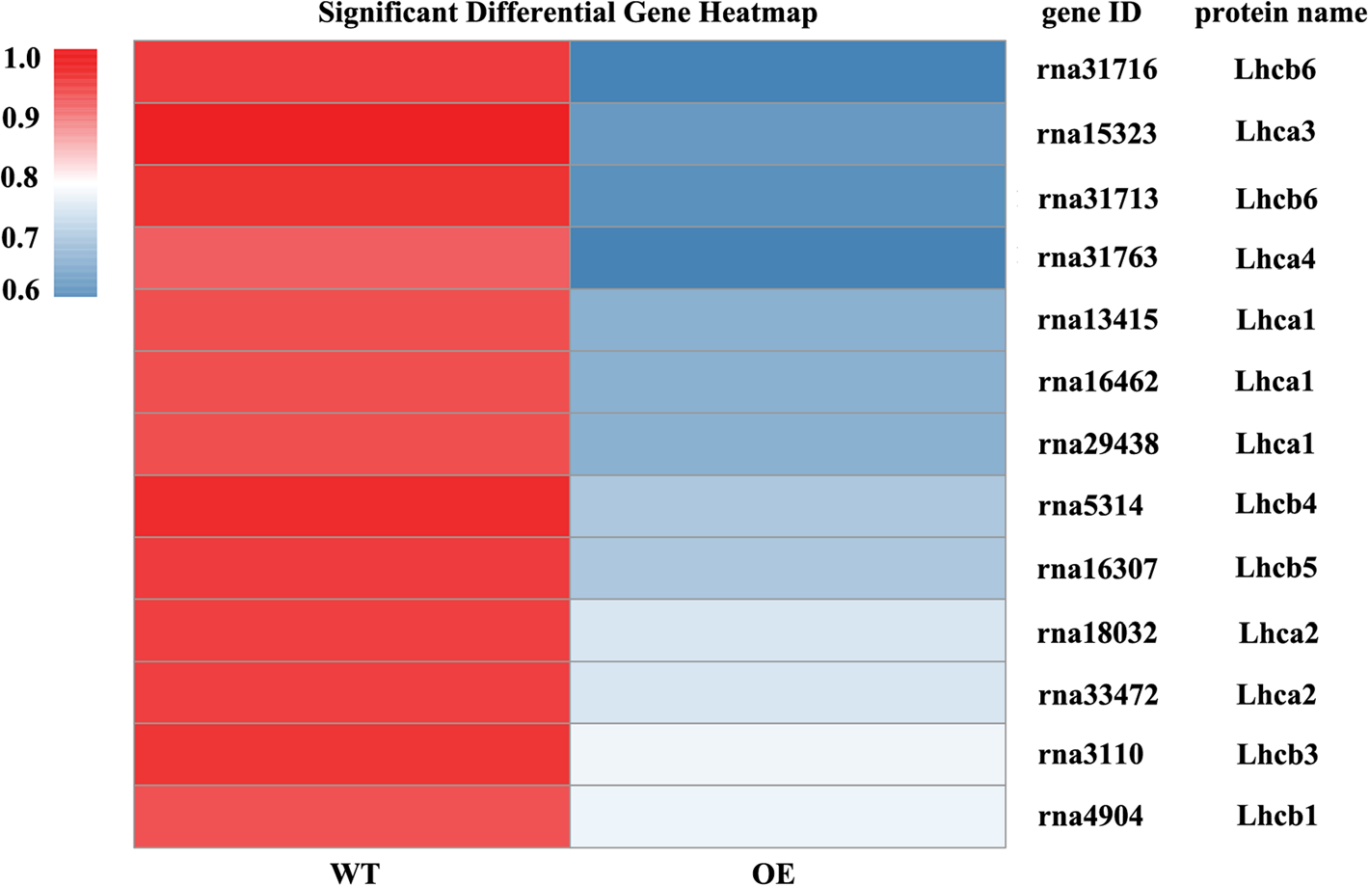
The up- or downregulated proteins in WT and OE line involved in “photosynthesis” pathway after cold treatment. **a** The proteins involve in “photosynthesis” pathway. Red color indicates upregulated proteins and green color indicates downregulated proteins. **b** Heat maps representation of differentially expressed protein. The bottom color bar indicates the fold change value for each differentially expressed protein. Red or blue color indicates upregulation or downregulation, respectively, at *P* < 0.05

**Fig. 10:**
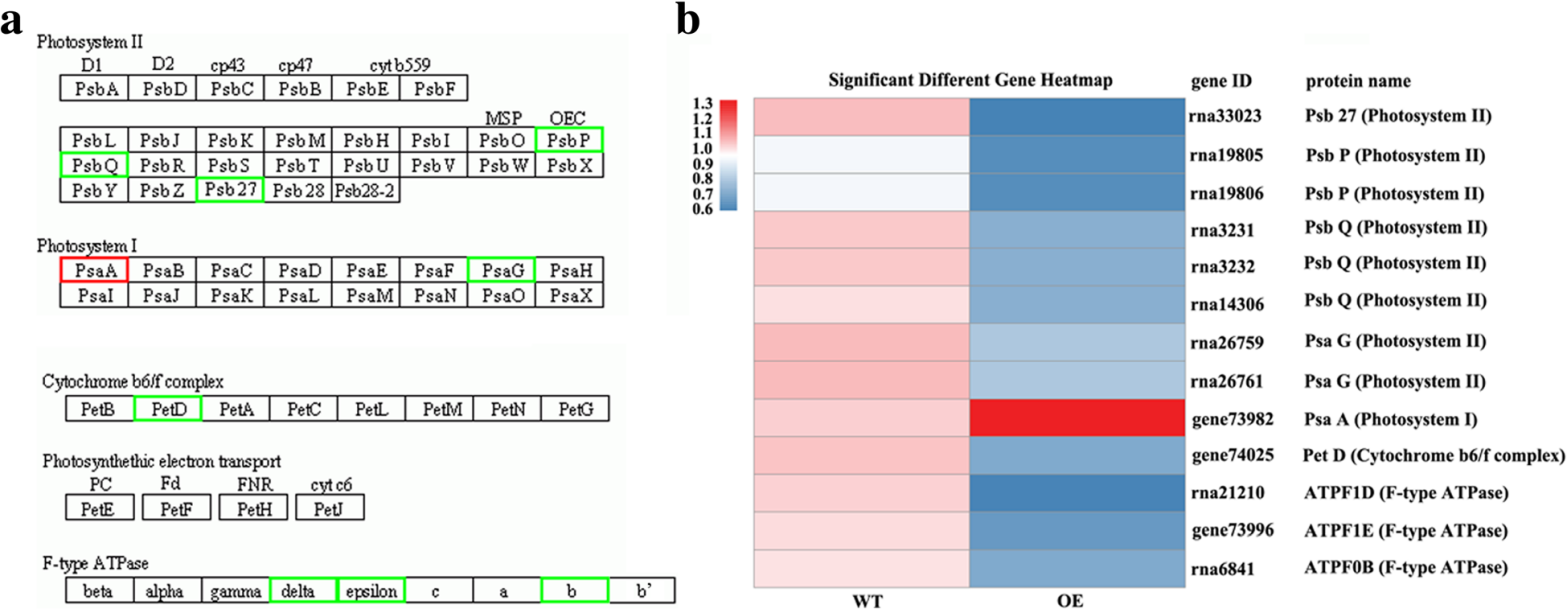
Heat maps showing the up- or downregulated proteins in WT and OE line involved in “photosynthesis-antenna protein” pathway after cold treatment. The bottom color bar indicates the fold change value for each differentially expressed protein. Red or blue color indicates upregulation or downregulation, respectively, at *P* < 0.05

**Fig. 11:**
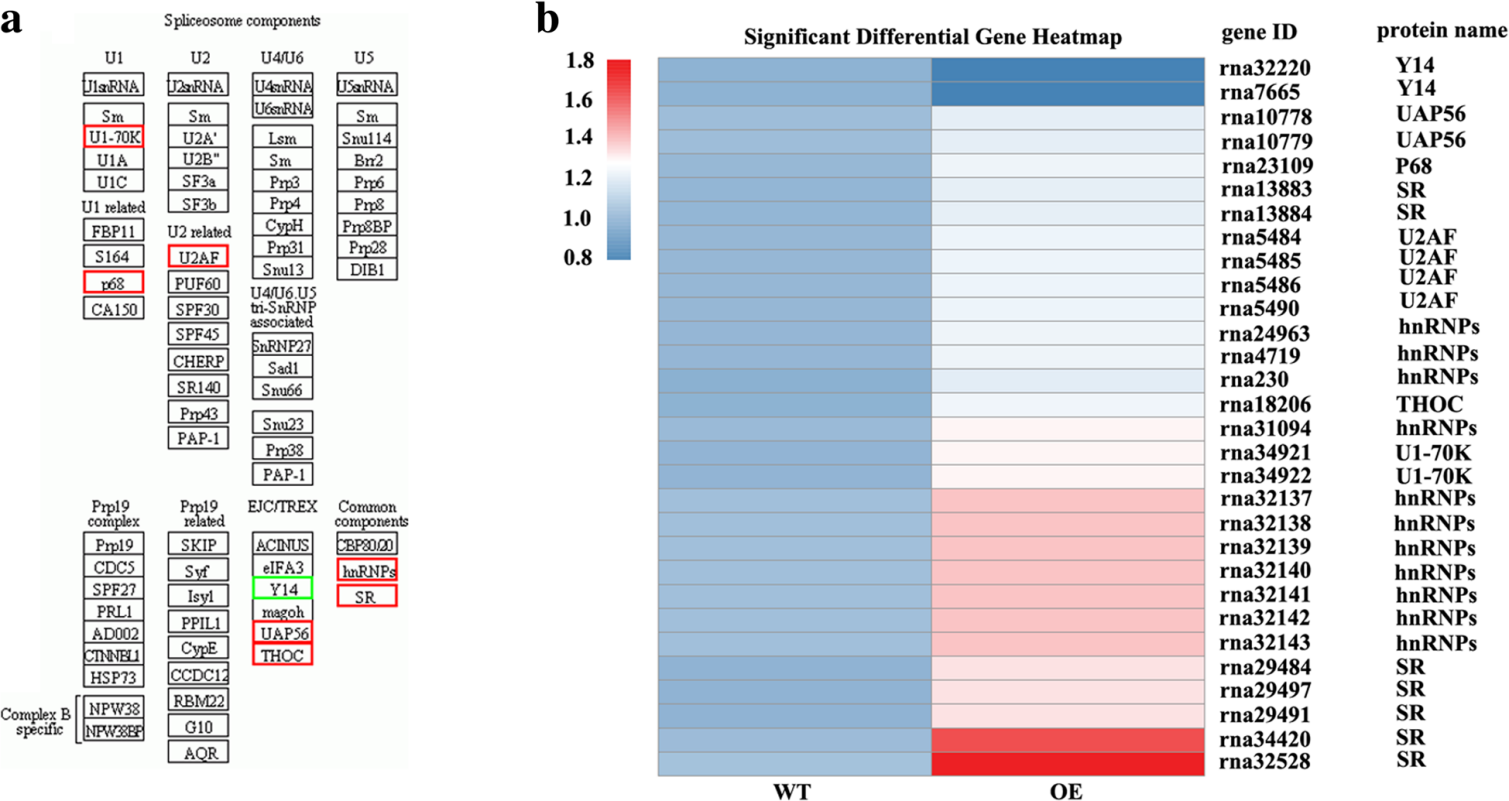
The up- or downregulated proteins in WT and OE line involved in “splicesome” pathway after cold treatment. **a** The proteins involve in “splicesome” pathway. Red color indicates upregulated proteins and green color indicates downregulated proteins. **b** Heat maps representation of differentially expressed protein (*P* < 0.05). The bottom color bar indicates the fold change value for each differentially expressed protein. Red or blue color indicates upregulation or downregulation, respectively

KEGG pathway enrichment analysis was carried out for further understanding the biological functions of DEPs. There were 160 DEPs being matched to KEGG pathway including 49 signaling/metabolic pathways. Most of DEPs were significantly enriched in pathways of “photosynthesis-antenna protein”, “spliceosome”, “photosynthesis”,“peroxisome”, “ribosome”, “DNA replication”, “RNA transport” and “phenylpropanoid biosynthesis” (Table 1). For example, a total of 26 DEPs were involved in “photosynthesis” and “photosynthesis antenna protein”. The DEPs (13) involving in “photosynthesis” pathway included one subunit of Psb27, PsaA, PetD (subunit IV of cytochrome *b*_*6*_*f*), ATPF1D (delta subunit of ATP synthase), ATPF1E (epsilon subunit of ATP synthase), and ATPF0B (b subunit of ATP synthase), two subunits of PsbP and PsaG, and three subunits of PsbQ. Most of them showed down-regulated in transgenic tobacco compared with the wild type, except for PsaA which was up-regulated (Fig. 9a, b). Thirteen DEPs annotated as “photosynthesis-antenna protein” or LHC included one subunit of Lhca3, Lhca4, Lhcb1, Lhcb 3, Lhcb4 and Lhcb5, two subunits of Lhca2 and Lhcb6, and three subunits of Lhca1. All of them were down-regulated in transgenic tobacco as compared with the wild type (Fig. 10). Thirty DEPs were grouped to “spliceosome” pathway. They belong to subunits of seven upregulated proteins including U1-70 K, p68, U2AF, UAP56, THOC, hnRNPs, and SR and subunits of one downregulated protein Y14 in transgenic plant as compared with the wild type (Fig. 11a, b).

**Table 1:** Significantly enriched pathways in the DEPs

Pathway	DEPs with pathway annotation (160)	All proteins with pathway annotation (1914)	*p-* value	Pathway ID
Photosynthesis-antenna proteins	13 (8.13%)	15 (0.78%)	0.000000	ko00196
Spliceosome	30 (18.75%)	101 (5.28%)	0.000000	ko03040
Photosynthesis	13 (8.13%)	51 (2.66%)	0.000172	ko00195
Peroxisome	7 (4.38%)	35 (1.83%)	0.022700	ko04146
Ribosome	7 (4.38%)	214 (11.18%)	0.027091	ko03010
DNA replication	26 (16.25%)	16 (0.84%)	0.038627	ko03030
RNA transport	4 (2.5%)	84 (4.39%)	0.042792	ko03013
Phenylpropanoid biosynthesis	7 (4.38%)	41 (2.14%)	0.049751	ko00940

## Discussion

### MfEF2 confers cold tolerance with altering proteomic profiling

A cold responsive *MfEF2* from *M. falcata* was identified in this study. MfEF2 showed a high similarity with other plant eEF2s including MtEF2 and AtEF2. Although our knowledge on the role of eEF2 in plant growth and development as well abiotic stress adaptation is limited, an early observation on *Arabidopsis* mutant *los1–1* reveals an essential role of AtEF2 in cold tolerance. One point mutation of Cys495 residue that is conserved in all plant eEF-2 proteins in *los1–1* resulted in reduced cold tolerance [24]. In this study, we found that *MfEF2* transcript was induced in response to low temperature, and overexpression of *MfEF2* resulted in enhanced freezing and chilling tolerance in transgenic plants. Compared to the wild type, transgenic tobacco plants had higher survival rate after freezing, or had lower levels of ion leakage and ROS accumulation and higher levels of *A* and *F*_v_/*F*_m_ after chilling treatment. Our results in combination with that in *los1–1* suggest that eEF2 confers cold tolerance and *MfEF2* is a potential candidate gene used for crop improvement of cold tolerance.

Eukaryotic translation elongation factor-2 functions to mediate the translocation step in peptide chain elongation during protein synthesis. Although protein synthesis in the mutant *los1–1* is not altered at room temperature, new proteins synthesis is blocked under low temperature [24]. In the present study, 336 DEPs including 181 up-regulated proteins and 155 down-regulated proteins were identified in a transgenic line as compared with the wild type after low temperature treatment, indicating that MfEF2 expression altered proteomic profiling under low temperature condition.

### Photosynthesis related proteins were regulated by expression of MfEF2 under low temperature condition

Photosynthesis is sensitive to low temperature [25]. Proteomics analysis revealed that many cold responsive proteins were related to chloroplast physiology and function in *Thellungiella*, rice and maize [26–28]. Light energy is captured and transmited by LHC rapidly to the photochemical reaction center of PSI and PSII located on thylakoid membranes to form energy carrying molecules ATP and NADPH [29], while ATP and NADPH are used for carbonhydrate formation during CO_2_ accimilation through Calvin-Benson cycle in chloroplast. In higher plants, LHCI in PSI antenna complex is composed of four different subunits named as Lhca1 to Lhca4 [30], while LHCII is composed of six different subunits (Lhcb1 to Lhcb6) in PSII antenna complexes [31]. All subunits of LHCI and LHCII except for Lhcb2 were down-regulated in transgenic plants as compared with the wild type under low temperature. The significant suppression of photosynthesis-related genes under cold stress has been observed in *Arabidopsis* and barley [32, 33]. Transcriptome analysis revealed that many genes encoding photosynthesis-antenna proteins (including Lhca1, Lhca2, Lhca4, Lhcb1, Lhcb4, Lhcb6) were significantly down-regulated in *Lotus japonicus* after cold treatment [34]. Expression of the genes encoding PSbP, PsbQ, PsbR, PsbW was suppressed in *Lotus japonicus* [35] in response to low temperature. Likely, PsbP, PsbQ, and Psb27, the subunits in the core complex of PSII, as well as PsaG, the subunit in the core complex of PSI, were down-regulated in transgenic plants compared with the wild type. The down regulation of LHC was an adaptation effect for reduced absorption of light energy by plants when light energy could not be used effectively under low temperature. The two photosystems are functionally connected by the plastoquinone (PQ) and cytochrome b6/f, which catalyze the building of the transthylakoid proton gradient that is dissipated for ATP synthesis by chloroplast ATP synthase [36]. The down-regulated expression of PetD, ATPF1E, ATPF1D and ATPF0B, which was consistent with that of LHC and core complex of PSII and PSI, would lead to decreased ATP synthesis in transgenic plants as compared with the wild type under low temperature.

Exposure to low temperature decreases activities of the enzymes involving in Calvin-Benson cycle, which leads to reduced utilization of absorbed light energy for CO_2_ assimilation and increased production of ROS via the water-water cycle [37]. Down regulation of the LHC proteins may lead to decreased capacity to capture sun light and reduced photochemical reaction in transgenic tobacco under low temperature conditions, which result in reduced production of ROS and oxidative injury on plant cells. This was confirmed by the less accumulated H_2_O_2_ in transgenic tobacco plants than in the wild type under low temperature. In addition, transgenic plants had higher levels of NPQ than the wild type. *NPQ* is one of the major photoprotective mechanisms to dissipate excitation energy absorbed by the light-harvesting antenna of photosystem II (PSII) as heat for protection of photosynthetic apparatus against damage [38]. Thus, the increased cold tolerance in *MfEF2* trasngenic plants was associated with the reduced expression of photosynthesis related proteins.

### MfEF2 regulates protein levels involving in splicesome under low temperature condition

A total of thirty DEPs involving in splicesome were observed in transgenic tobacco as compared with the wild type in the present study. The DEPs belong to subunits of seven upregulated proteins including U1-70 K, p68, U2AF, hnRNPs, SR, UAP56, and THOC and one downregulated protein Y14. Spliceosome is a multi-megadalton ribonucleoprotein (RNP) complex catalyzing nuclear pre-mRNA splicing. Alternative splicing is frequently associated with regulation of cold responsive genes in plants [39, 40]. A pre-mRNA splicing factor *STABILIZED1* (*STA1*) in *Arabidopsis* is strongly upregulated by cold stress, while the mutant *sta1* shows mis-splicing of RNAs under cold stress with extremely cold sensitive, suggesting that pre-mRNA splicing is essential for cold tolerance [41]. The serine/arginine-rich (SR) proteins are involved in splicing control by promoting alternative splicing of their own transcripts as well as other gene products [42]. Most of the mRNAs of SR genes in *Arabidopsis* undergo alternative splicing following cold, heat, salt stress [40, 43, 44]. In addition, alternative splicing of the circadian clock gens depends on temperature, which is associated with the interaction of the conserved SNW/Ski-interacting protein (SKIP) domain-containing proteins with the spliceosomal splicing factor SR45 [45]. Nevertheless, our data suggested that the enhanced cold tolerance in *MfEF2* trasngenic tobacco plants is associated with splicesome. However, the information on alternative splicing profiles and the target splicing genes responsive to cold as affected by MfEF2 remain to be further investigated. A genome-wide analysis of alternative pre-mRNA splicing between the wild type and *MfEF2* overexpression plants based on full-length cDNA sequences will be conducted in the future.

## Conclusions

*MfEF2* transcript was induced in response to low temperature in *M. falcata*. Overexpression of *MfEF2* resulted in enhanced cold tolerance with reduced production of ROS. Hundreds of DEPs in transgenic plants versus the wild type under low temperature were enriched in multiple pathways, while many of them are involved in photosynthesis as subunits of PS I and PSII as well as LHC showing downregulation and spliceosome showing upregulation in transgenic plants as compared with the wild type. It is suggested that the enhanced cold tolerance in *MfEF2* transgenic tobacco was associated with downregulation of photosynthesis related DEPs, which leads to reduced capacity to capture of sun light and photochemical reaction but increased *NPQ* to dissipate excitation energy as heat and thus results in reduced ROS production for protectin of plants against oxidative injury under low temperature, and upregulation of the DEPs involving in splicesome, which promotes alternative splicing of pre-mRNA of cold responsive genes.

## Methods

### Plant growth

*Medicago falcata* and the homozygous lines (T_3_) of transgenic tobacco plants overexpressing *MfEF2* in combination with the wild type were grown in 15-cm diameter plastic pots containing a mixture of peat and perlite (3:1, v/v) in greenhouse with temperature ranging from 20 to 28 °C under natural light. Two-month-old *M. falcata* plants were used for cold treatment at 5 °C for 4 d for gene expression in response to cold, while two-month-old tobacco plants were used for freezing or chilling treatment for assessment of cold tolerance. *Medicago falcata* seeds were sent from Chinese Academy of Agricultural Sciences (Beijing 100,193, China), while the seeds of tobacco were reproduced and kept in our laboratory after they were originally sent from Guangdong Academy of Agricultural Sciences (Guangzhou 510,641, China).

### RT-PCR and qRT-PCR

Total RNA was extracted using TRIzol reagent (Invitrogen) according to the manufacturer’s instructions. The cDNA was synthesized from 1 μg of total RNA, using the PrimeScript RT reagent Kit with gDNA Eraser (Takara, Japan). For cloning of *MfEF2*, forward primer RT77 (5′-CTAGTCAAGATGGTGAAGTTCACAG-3′) and reverse primer RT78 (5′-CAGTTTTCATAACAGCCAAGTACAT-3′) were designed after an assembly of EST sequences using SeqMan (DNASTAR Inc., Madison, WI, USA) based on *EF2* sequence data from the GenBank. RT-PCR was conducted in a reaction mixture containing the cDNA, dNTPs, primers RT77 and RT78, and *Ex Taq* DNA polymerase (Takara, Dalian, China). *MfEF2* cDNA and the deduced amino acid sequences were analyzed using DNAMAN software (Lynnon Biosoft, Vaudreuil, Quebec, Canada). *EF2* transcript was analyzed uisng qRT-PCR in MiniOption Real-Time PCR System (Bio-Rad, Hercules, CA) following the manufacturer’s instructions. The PCR reaction mix was consisted of diluted cDNA as template, 200 nM forward and reverse primers, and 5 μl SYBR Premix *Ex Taq* (Takara, Dalian, China). Parallel reactions to amplify *actin1* were used to normalize the amount of template. ZG1753 (5′-GCACTCCGTATTACTGATGG-3′) and ZG1754 (5′-TCTGGTATGCCTCCTCTC-3′) were used as forward and reverse primers of *MfEF2*, while ZG1613 (5′-ATTCACGAGACCACCTAC-3′) and ZG1614 (5′-GAGCCACAACCTTAATCTTC-3′) were used for amplification *actin* respectively. Relative expression was calculated by 2^-ΔΔCt^. The primer specifcity was validated by melting profles, showing a single product specifc melting temperature. All PCR effciencies were above 95%.

### Transgenic tobacco generation and DNA and RNA blot hybridization

The coding sequence of *MfEF2* was cloned into the pBI121 binary vector to construct an expressing plasmid pBI-*MfEF2*. Transgenic plants was generated using *Agrobacterium tumefaciens*-mediated transformation after selection by kanamycin as previously described [11]. Gnomic DNA was isolated using CTAB. Ten μg was used for digestion with *Xba* I overnight (Takara Bio Inc*.* Dalian, China) for DNA blot analysis, while 20 μg total RNA was used for RNA blot analysis. *NPTII* and *MfEF2* gene were labeled as probe using [α-^32^P] dCTP for DNA and RNA blot hybridization, respectively, using the standard procesure. Hybridization signals were detected on Typhoon Trio (General Electric Company, Fairfield, CT).

### Cold tolerance assay

Survival rate was determined to evaluate freezing tolerance as described previously [17]. Fifteen to twenty two-month-old tobacco plants in each pot were placed in a growth chamber with temperature decreasing from 25 to − 3 °C linearly within 6 h and maintained for 10 h under light of 700 μmol photon m^− 2^ s^− 1^. Five pots of plants were employed as replicates in each experiment. Plant survival rate was calculated, and photos were taken 5 days after plants were moved to room temperature for recovery. For chilling treatment, tobacco plants were placed in a growth chamber at 2 °C for 3 d with a 12-h photoperiod under light of 700 μmol photon m^− 2^ s^− 1^. Ion leakage was measured by reading conductivity (*C1*) 12 h after placing leaflets in to tubes and followed by addition of 10 ml deionized water in each tube. Tubes were then heated in a boiling water bath for 20 min before measuring the total potential conductivity (*C*2) at room temperature. The percentage of ion leakage was calculated as (*C*1/*C*2) * 100. *F*_v_/*F*_m_, *q*_p_ and *NPQ* were measured from five plants of each line using a pulse-modulated fluorometer (Model FMS-2, Hansatech Instruments) according to the manufacturer’s instructions [46], while net photosynthetic rate (*A*) was determined from ten plants using using portable photosynthesis system (model LI-6400; LI-COR Biosciences, Lincoln, NE) according to the manufacturer’s instructions [14].

### H_2_O_2_ detection

Hydrogen peroxide was visualized by staining with 3, 3-diaminobenzidine (DAB). Plant leaves were excised and supplied with solution of 1 mg/mL DAB in Tris-HCl buffer (pH 6.5) containing 0.01% Triton X-100. The samples were incubated at room temperature for 1 h in the dark, followed by decoloring in boiling ethanol for 20 min as described previously [17].

### Protein extraction, digestion, iTRAQ labeling and LC-MS/MS analysis

Total proteins were extracted using the cold acetone method. Leaves were sampled from the transgenic line (54) and the wild type after treated for 48 h at 3 °C. The leaf samples were powdered with liquid nitrogen, followed by extraction in Lysis buffer consisting of 8 M urea, 2% SDS and 1× Protease Inhibitor Cocktail (Roche Ltd. Basel, Switzerland) with sonication on ice for 30 min. The homogenate was centrifuged at 12000 rpm for 20 min at 4 °C. The supernatant was moved into a fresh tube and incubated at − 20 °C overnight, followed by centrifugation at 25000 g for 20 min. The precipitate was suspended in pre-cooled acetone and centrifuged as above. This procedure was repeated until the supernatant was completely colorless. After air-dried, the precipitate was incorporated into 8 M Lysis buffer, incubated at room temperature with sonication for 15 min, and then centrifuged as above. Protein quality and concentrations were determined with SDS-PAGE and the Bradford assay. Two biological replicates were prepared for the iTRAQ analysis, which was conducted (Gene Denovo Biotechnology Co., Guangzhou, China) as described before [46].

Protein was digested with Trypsin Gold (Promega, Madison, WI, USA). After the digested samples were dissolved in 500 mM Triethylammonium bicarbonate (TEAB) solution. The wild type and transgenic tobacco plant samples were labeled with iTRAQ tags according to manufacturer’s protocol in the iTRAQ 8-plex labelling kit (Applied Biosystems, Foster City, CA, USA). The labeled samples were pooled and dried by vacuum centrifugation.

The iTRAQ-labeled peptide mixtures were eluted and then fractionated by high pH separation. The peptides were eluted as follows: from 5 to 45% buffer B (20 mM ammonium formate in 80% ACN, pH 10.0) in 40 min. Fifteen fractions were collected and dried in a vacuum concentrator. Peptide fractions were resuspended in 30 μl solution C (water with 0.1% formic acid), separated by nanoLC, and analyzed by on-line electrospray tandem mass spectrometry. The experiments were performed on an Easy-nLC 1000 system (Thermo Fisher Scientific, MA, USA) connected to a Orbitrap Fusion Tribrid mass spectrometer (Thermo Fisher Scientific, MA, USA) equipped with an online nano-electrospray ion source.

The fusion mass spectrometer was operated in the data-dependent acquisition mode to switch automatically between MS and MS/MS acquisition. Full-scan MS spectra (m/z 350–1550) were acquired with a mass resolution of 120 K, followed by sequential high energy collisional dissociation (HCD) MS/MS scans with a resolution of 30 K. The isolation window was set as 1.6 Da. The AGC target was set as 400,000. MS/MS fixed first mass was set at 110. In all cases, one microscan was recorded using dynamic exclusion of 45 s.

### Protein identification and quantification

Raw data files from LC-MS/MS were converted into MGF (MASCOT generic format) files with Proteome Discovery 1.2 (Thermo, Pittsburgh, PA, USA). Proteins were identified using the Mascot search engine (Matrix Science, London, UK; version 2.5.1). Mascot database was set up for protein identification using *Nicotiana tabacum* reference transcriptome (https://www.ncbi.nlm.nih.gov/genome/?term=tobacco). Protein quantification was carried out in those proteins identified in all the samples with unique spectra ≥2. The Mascot search results were averaged using medians and quantified. Proteins with fold change in a comparison >1.2 or <0.83 and unadjusted significance level *P* < 0.05 were considered differentially expressed [47].

### Bioinformatics analysis

Differentially expressed proteins were classified and identified using the Gene Ontology (GO) (http://www.geneontology.org) and the Kyoto Encyclopedia of Genes and Genomes (KEGG) (http://www.genome.jp/kegg/ or http://www.kegg.jp/). Significant pathway enrichment was examined with the hypergeometric test. A *P*-value ≤0.05 was used as the threshold to determine the significant enrichments of GO and KEGG pathways.

## Additional files

Additional file 1: **Figure S1.** Basic information statistics of Protein identification (PDF 5 kb) (PDF 4 kb)

Additional file 2: **Table S1.** List of the total 2939 proteins identified in this study (XLS 3170 kb) (XLS 3169 kb)

Additional file 3: **Figure S2.** Mass distribution of the identified protein (PDF 5 kb) (PDF 4 kb)

Additional file 4: **Table S2.** List of upregulated and downregulated proteins identified and quantified by iTRAQ analysis in transgenic plant versus the wild type (XLS 469 kb) (XLS 468 kb)

## Abbreviations

*A*: Net photosynthetic rate
CBF: CRT binding factor
COR: Cold regulated
DEPs: Differentially expressed proteins
eEF-2: Eukaryotic translation elongation factor-2 (eEF-2)
ERF: Ethylene responsive factor
*F*_v_/*F*_m_: Maximum photochemical efciency of PSII
GolS: Galactinol synthase
INT-like: Myo-inositol transporter-like
iTRAQ: Isobaric tags for relative and absolute quantification
LHC: Light harvesting chlorophyll protein complex
MIPS: Myo-inositol phosphate synthase
*NPQ*: Nonphotochemical quenching
ORF: Open reading frame
PIP: Plasma membrane intrinsic protein
PS: Photosystem
*q*_p_: Photochemical quenching
ROS: Reactive oxygen species
SAMS: S-adenosylmethionine synthetase
SSH: Suppression subtractive hybridization
TIL: Temperature induced lipocalin
