# Supplementary figures and images for: An eukaryotic elongation factor 2 from Medicago falcata (MfEF2) confers cold tolerance
Source: BMC Plant Biol. 2019 May 27;19:218. doi: 10.1186/s12870-019-1826-7 (PMC6537394; doi:10.1186/s12870-019-1826-7)

# basic information statistics

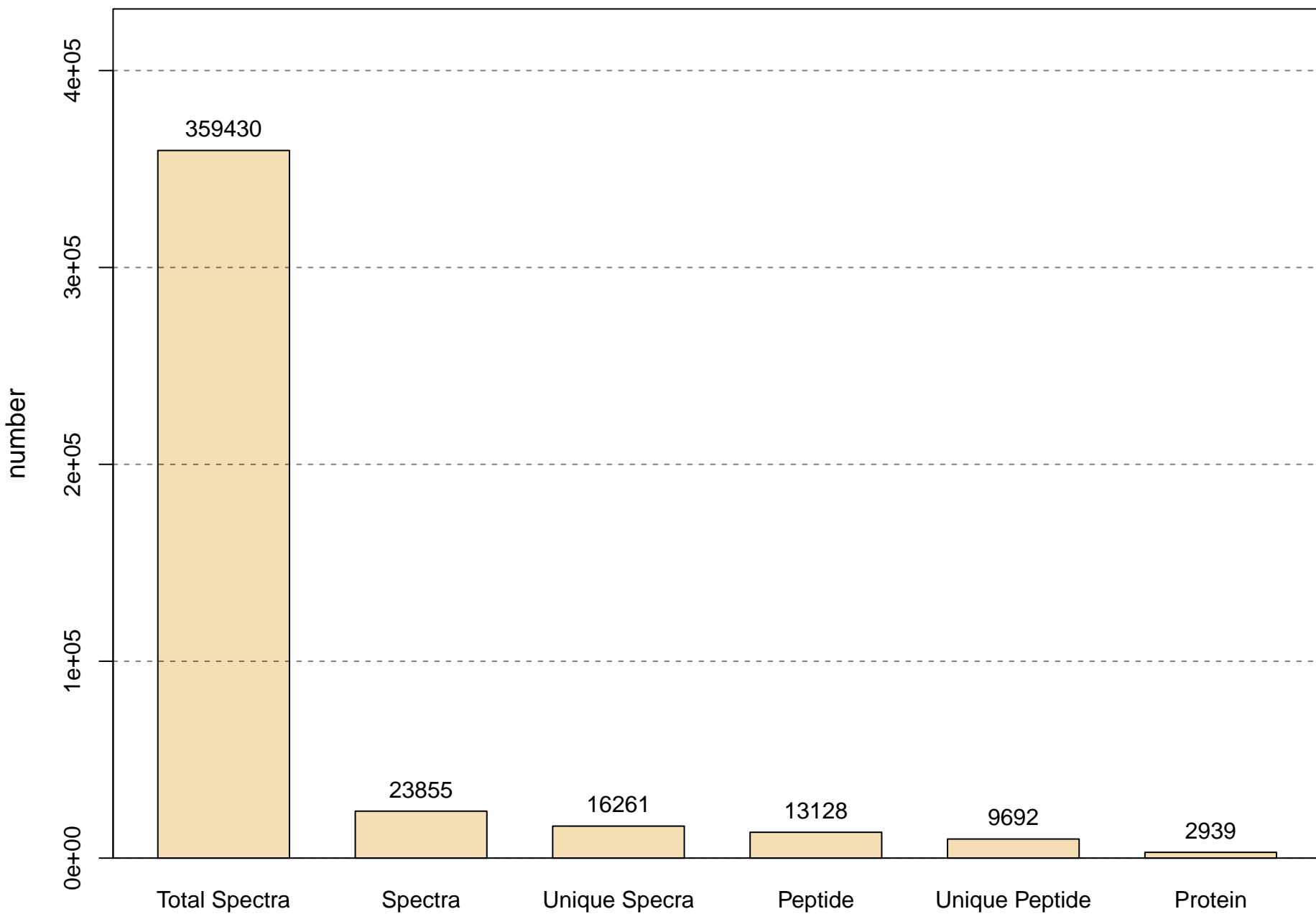

Supplement: Supplementary file 1 — Figure S1. Basic information statistics of Protein identification (PDF 5 kb) (PDF 4 kb) [file 12870_2019_1826_MOESM1_ESM.pdf]

# protein mass distribution

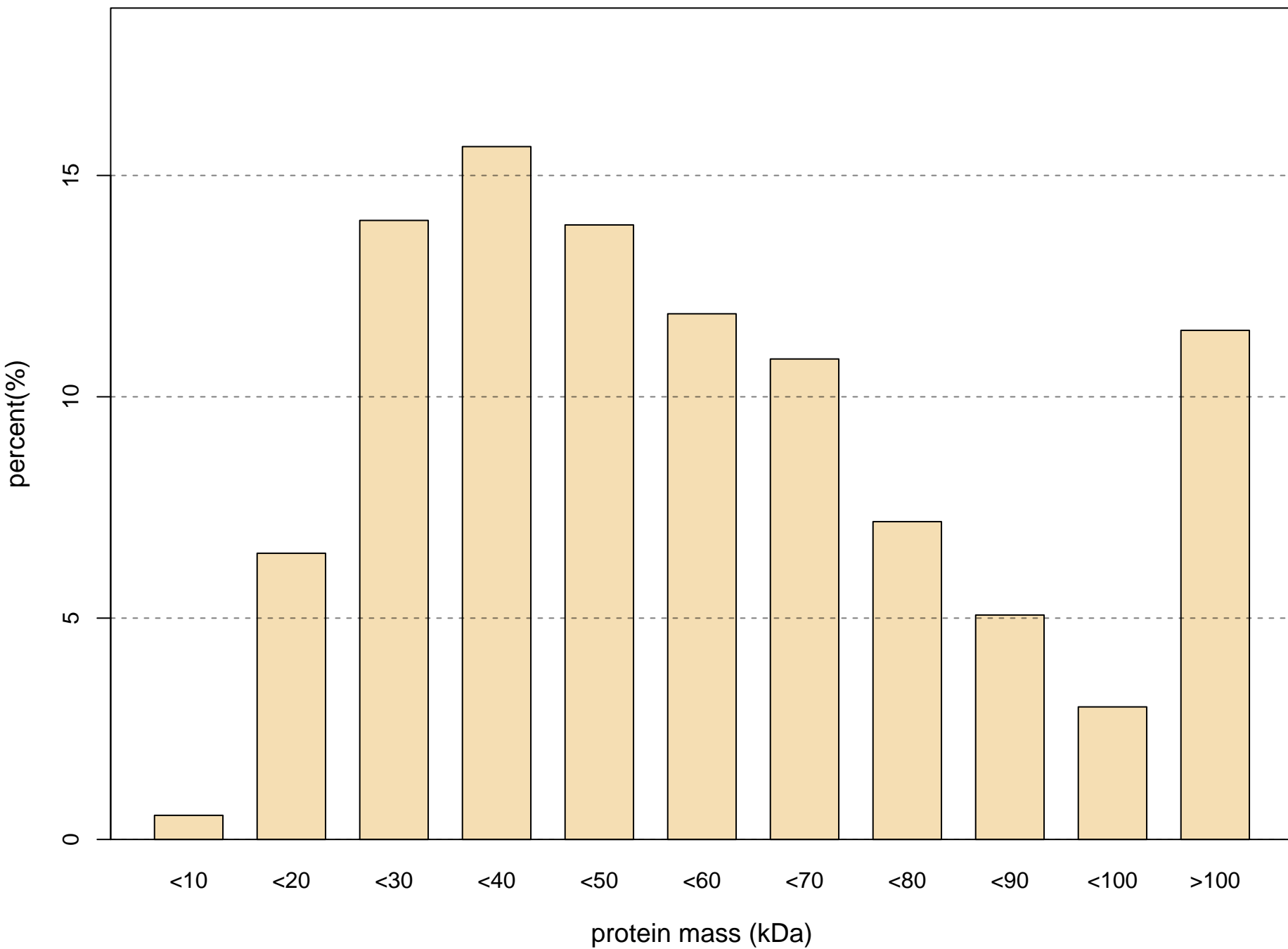

Supplement: Supplementary file 3 — Figure S2. Mass distribution of the identified protein (PDF 5 kb) (PDF 4 kb) [file 12870_2019_1826_MOESM3_ESM.pdf]
